# Abstracts and Gleanings

**Published:** 1887-02

**Authors:** 


### ABSTRACTS AND GLEANINGS.

**Letter from Germany.**—Dr. Littig, correspondent *Iowa State Medical Reporter*, writes .from Berlin, October 27, 1886: "*Dear Reporter*—Thinking that a few words regarding the methods of Prof. Bergmann, of the Berlin University, might be of interest to you, I write you a short letter.

"Over fifteen thousand cases are treated each year at the Royal Clinic and Policlinic, and from this superabundant material, cases are selected for operation before students. During the semester the professor operates two hours daily. In the management of this hospital, there is but one mind—one man whose wishes are consulted—viz.: Bergmann. There is no board to stand between him and the relization of his ideas of a perfect hospital. Skilled assistants of his own choosing aid him in the operating room, while nurses of his own training are in constant attendance in the wards. But it is of the operating room that I wish to speak. To produce anæsthesia, chloroform only is used; a single thickness of flannel cloth stretched over a wire frame is the inhalation apparatus, and upon this cloth, placed over the patient's mouth, the anæsthetic is dropped—not poured. Take a three-ounce vial, fit the stopper with two glass or metal tubes, one reaching to the bottom for the admission of air, the other just through the stopper to permit escape of fluid, as precisely as in a druggist's wash bottle. The finger over the external opening of the air-admitting tube permits the chloroform to escape in a very minute stream, or drop by drop, as willed by the anæsthetizer. It may not be amiss to state that Professor Schrœder and Bardeleben also uses chloroform administered in the same way. As soon as the patient is partially under the influence of the anæsthetic, a very important part of the operation begins. An assistant washes the part to be operated on, scrubs it with soap and brush, sparing neither soap nor friction. Next, the copious lather thus produced is washed off with a one-to-one-thousand solution of bichloride of mercury. The parts around the seat of the operation are covered with one or more towels, lightly wrung out of the same solution, and should the operator not be ready, several folds of gauze, also dipped in bichloride solution, are spread over the part to be operated on. The operator, as all assistants, has thoroughly washed and disinfected his hands, and all are clothed in white, newly washed, fully disinfected operating gowns, which reach to the ankles. The instruments, all with metal handles, are lying in a three-to-one-hundred solution of carbolic acid.

"To control hemorrhage, Esmarch's rubber bandage is not used; it is exceedingly unclean from an antiseptic standpoint, and so bruises the tissues that subsequent capillary oozing is far more profuse with than without its use. Instead, the arm or leg is raised for an instant, stroked in that direction of the body to encourage the return of venous blood, and a piece of rubber tubing is passed one or more times around the limb, near its junction with the body.

With this precaution there is absolutely no loss of blood—no bleeding until the rubber is loosened. Should the operation be one in which the '*vis. a tergo*' cannot be controlled, as the excission of the mamma, assistants at once catch up every bleeding point with an ordinary slide-catch torsion forceps. As many as twenty of these instruments may be bristling from a wound before they stop to ligate with cat-gut. Every point that has bled is tied—*i. e.* they don't remove a forcep to see if bleeding still continues, but at once ligate everything, differing in this respect from the practice at Guy's, London, where they make it a rule not to ligate even the femoral, torsion taking the place of the ligature in all cases. In some operations they may stop several times to ligate, when the forceps grow too numerous. The spray is not used. From time to time during a long operation the wound is rinsed out with a solution of bichloride, and always before closing. Carbolic acid does not come in contact with the wound, except when the saw is used, when a gentle stream of a three-to-one-hundred solution is thrown, by means of a fountain syringe, against the teeth of the saw, thus securing a double end disinfection and the saw runs more easily.

"All incised wounds are closed at once, unless so situated that pocketing is imminent, when a drainage tube is introduced. Some wounds, as a psoas abscess, are packed for three days with iodoform gauze, when the gauze is removed, a drainage tube is introduced and the wound closed. The same practice is pursued in an exsection of the ankle joint. Iodoform gauze is prepared by saturating strips of gauze with the following solution, and subsequently drying:

| | |
|---|---|
| Iodoform | 1 part |
| Ether | 2 parts, |
| Alcohol | 4 parts. |

Where it is desirable to pack a wound, gauze prepared in this way is certainly a most excellent dressing. When a wound has been closed by means of sutures or over the iodoform gauze packing, the dressing is as follows: First, antiseptic gauze, not applied in so many layers, but it is cut in squares as large as a lady's handkerchief, and being crumpled, is merely piled over and about the wound. Next comes antiseptic absorbent cotton. This is cut in strips about four inches wide, and applied not only over the wound, but for some distance above and below, and around the entire body. For instance, in the incision of the knee, this cotton is applied from the toes to the umbilicus. Over the cotton an ordinary roller bandage, and over all the starch bandage. This starch badage is purchased in supply stores, and before use is soaked in water as is our plaster bandage, and applied wet. It is light, holds all under-dressings firmly in place and secures a certain amount of immobility. Of course, where absolute fixation is demanded, as in knee excision, this starch bandage is replaced by one of plaster Paris.

"The above dressing is certainly almost perfect. The gauze and cotton are prepared by soaking in the following solution, and subsequently drying:

Bi-chloride (of a ten per cent. solution)...... 7 parts,
Alcohol.......................................1000 parts,
Water.........................................1000 parts,
Glycerine.....................................400 parts.

"Bergmann operates carefully, fearlessly, in the full sense of the word. During a long period of visits at his clinic, one sees many excisions, but few amputations. In knee-joint he saws through and retains the patella.

"By way of closing, it may be interesting to note that a patient died from the effect of chloroform last semester, at Bardeleben's clinic. The autopsy revealed a fatty heart, and perhaps the fault was not so much that of the anæsthetic as the anæsthetizer, who should know the danger.

"Once I saw very dangerous symptoms at Bergmann's clinic. Artificial respiration for five minutes and all was well."

**Hot Bath.**—Sometime ago an opportunity was afforded me of making some observations on the effect of the hot bath in removing some morbid conditions of the system. A man of middle life, in temperament nervous-sanguine, spare, and somewhat below the average height and weight, complained of languor, debility, want of energy, and lowness of spirits. On examination it was found that his heart and arteries were sound, though his circulation was rather weak. His alimentary system was fairly good, though the quantity of food taken was below the average. His skin was somewhat dry, and a few spots of psoriasis were found on the extensor aspect of the legs, arms, and trunk. His urine was cloudy with phosphates, and below the average in quantity. As the Turkish bath was not in this case available, he was advised to take a water bath at a temperature of 105° F., and directions were given him how to proceed in case of faintness. The day after taking the bath his condition was wonderfully improved. His circulation was stronger, his urine was clear, and he now felt cheerful and well. This improved state of matters continued for about twelve days, when all the unpleasant symptoms reappeared, and he began to feel as ill and dejected as ever. The most natural proceeding was, of course, to order another bath, and this he took with the same happy results as before. Since that time he has had a hot bath about once a fortnight, and by this means has managed to keep himself in very tolerable health and spirits.

It would be interesting to know the exact means by which the hot bath produces this powerful and beneficial effect on the system. It must in the first place, be carefully borne in mind that the warm bath at a temperature below 100° F. has no influence that that can be compared with the results to be obtained from the hot bath. The hot bath stimulates the whole system, and the effect of this stimulation appears to last sometime, for the patient above referred to remained well for about a fortnight after taking the bath. Having ascertained that his diet was as nearly uniform as possible, the quantity of urea excreted on three successive days immediately before and after the bath was estimated by Russell and West's method, with the following results:

| | |
|---|---|
| Average quantity of urine in twenty-four hours for three days *before* the bath.. | 916.67 cc. |
| Average weight of urea in twenty-four hours for three days *before* the bath... | 22.8 grms. |
| Average quantity of urine in twenty-four hours for three days *after* the bath.... | 1040 cc. |
| Average weight of urea in twenty-four hours for three days *after* the bath.... | 26 25. |

As in most other remedies, so in the case of the hot bath, the time and manner of application are of some importance. Like all other baths, the hot bath should be taken before the principal meal of the day, and if this meal be taken early the good results will be more certain than if it be taken late. The duration of the bath should be about fifteen minutes, and the temperature should be about 105° F. If faintness should come on while in the bath, the whole head should be immersed in the hot water, and kept there for a few seconds, when the faintness will disappear. The usual directions given in public baths are to get out of the bath as soon as drowsiness or faintness begins, and ring for the attendant; but anyone who attempts to do this will most certainly aggravate his danger. As pointed out sometime ago by Mr. Benham and subsequently by myself, the application of heat to the head is a potent means of averting syncope. From time to time we hear of deaths in the warm bath; and I am convinced that many of these might have been prevented by the adoption of the simple method referred to, instead of the deadly and often impossible means commonly recommended.—*Dr. W. J. Notely, in London Lancet.*

**Solanine—A Substitute for Morphine.**—The many disadvantages of opium and its alkaloids are too well known to require mention, and while they are not sufficient to counterbalance the good effects of these drugs, they are of enough importance to make equally efficacious and more innocuous substitute a desideratum. According to Dr. Geneuil (*Bulletin General de Therapu-tique*, September 30, 1866), we have such a substitute in solanine, which is not a newly discovered remedy, but rather one which has fallen, the author thinks, into unmerited disuse.

Solanine was discovered by Defosses in 1821, in the berries of the nightshade (*Solanum nigrum*), and has since been found by others in the stems, and leaves, and berries of the bitter-sweet (*S. dulcamara*), and other varieties of solanaceæ. It is now prepared from potatoes, by boiling the young shoots in water slightly acidulated with sulphuric acid, and adding ammonia to the warm decoction. It can also be obtained from the parings of very young or very old potatoes. The alkaloid crystallizes in fine silky needles, insoluble in water, and but slightly soluble in ether, oils, and cold alcohol, but dissolving more readily in hot alcohol. It has an acrid taste, and imparts a burning sensation when placed in contact with the mucous membrane of the mouth.

The author has tried solanine in a number of cases of neuralgia, rheumatism, obstinate vomiting, spasmodic nervous affections, asthma, and bronchitis, and from the results obtained is led to believe that the remedy will prove to be of the greatest value in the treatment of these and similar affections. The following are the conclusions of his paper:

1. Solanine is a poison to the terminal motor plates. It narcotizes the medulla and spinal cord, causing a paralysis of the terminal sensory, and motor nerves. By reason of this action solanine is to be classed among the best of the analgesics.

2. The drug may be prescribed in large doses without danger, and presents none of the inconveniencies of morphine or atropine. There is no danger of a cumulative action.

3. Solanine does not cause congestion of the brain, even in the aged, and, probably, a like freedom from this danger exists in the case of children.

4. In all cases where it is necessary to calm excitement, relieve pain, or overcome spasm, solanine promises excellent results. It may be given with advantage in the place of morphine for the relief of any of these conditions.

If the author's exalted opinion of this remedy is borne out by further experience, it will indeed prove to be one of the most valuable of recent additions to the materia medica. But medicine is an uncertain science, and others may not meet with such wonderful success as Dr. Geneuil himself has.—*London Lancet.*

**Fracturcd Femur.**—Dr. A. C. Graham (*Texas Courier-Medicine*) thus describes the application of Paris bandage in fractured femur:

"The patient is placed upon the table, the sacrum resting on the tripod, the upper part of the body raised to a corresponding level; the feet bandaged to the foot pieces, and the double perineal band adjusted. Ether is now given, and extension used till both lower limbs are the same length. The cotton batting having been cut to fit the limb and body as high as the umbilicus, or higher is recommended by Prof. Cowling, by cotton thread. The badages, soaked well in water, are run from the ankle up to the leg, spirally or figure eight fashion, three or more successive layers—a paste of freshly mixed plaster being rubbed smoothly by the assistant over each bandage so as to assure additional strength to amalgamate the whole. As soon as the perineum is reached, protect the plaster by the oil silk, and then proceed with the spica, carrying it six inches above the crest of the ilium. The plaster will harden sufficiently in half an hour for the extension to be removed and the patient placed in bed.

"A few points call for additional mention. The plaster-bandage contracts as it dries, so that great care must be used not to apply it so tightly as to constrict the limb, or interfere too much with the limb, or interfere too much with the blood supply, as Brodie has shown that this retards union.

"The ankle and perineum must be protected by a double thickness of cotton batting. Guard against eversion of the leg; it were better that the foot be turned a little in, pigeon-toe fashion. The bowing outward, when the fracture is above the middle, must be contracted by forcible pressure against the splint in an opposite direction as the plaster is setting. As the spica is being applied, the plaster casing must be moulded by pressure, to all the inequalities of the hip, pelvis and lower abdomen, in this way securing a perfect cast.

"For a few days it is necessary to see the patient often—to note the circulation of the blood in the toes—to obviate any undue pressure and to make the patient as comfortable as possible. 'Eternal vigilence is here the price of success.'

In two weeks the plaster-case will be so loose that it will be necessary to remove it and put on another. No attempt should be made to narrow and tighten the old one.

"I have purposely avoided quoting statistics that have been brought forward to prove the superior efficacy of any one of these appliances in the prevention of shortening. They only show that good results have been obtained with all of them. An accurate estimate of the degree of shortening can seldom be arrived at for two reasons:

"1. It is now well known that there is normally a difference in the length of the lower extremities, this difference sometimes amounting to as much as three-fourths of an inch.

"2. The degree of shortening obtained when the apparatus is removed, is increased somewhat when the patient begins to walk

"I claim, however, for the plaster of Paris bandage, superiority in the following particulars and that for these we are justified in expecting better results in practice:

"1. It is the rational treatment, in that after the fracture is adjusted, perfect fixation of the fragments is obtained.

"2. It exerts a gentle, even compression of the whole thigh, thus moulding, as it were, the parts into their normal position.

"3. The hip-joint is completely immobilized.

"4. Angular deformity, or bowing outward, when the fracture is in the upper half, is prevented by compression of those muscles that abduct and rotate the thigh outward; also by forcible inward pressure on the splint, at the angle, as the plaster sets.

"5. We have perfect control of lower fragment and leg, and rotary displacement, or eversion, cannot take place.

**Suprapubic Cystotomy.**—From the proceedings in the section of surgery of the British Medical Association, we learn that Sir Henry Thompson throws the full weight of his valuable opinion in favor of suprapubic, as against the other methods of cystotomy.

For the severe and exceptional conditions he considers the suprapubic operation an available and trustworthy resource.

Its superiority over the lateral mode is thus formulated:

1. Because in the suprapubic operation there are no important structures lying in the line of incision, or sufficiently near to be rendered liable to injury either by the knife or by the forceps.

2. Because the space for removing a large stone above the pubes is practically unlimited.

3. Because there is little or no danger from hemorrhage; if it does occur, it can readily be dealt with.

4. Because the incisions are certainly more easy to perform than those of lateral lithotomy; while the removal of a large stone, always the most difficult and dangerous part of the operation, is safe and easy by the suprapubic route.

5. Because, during the after-treatment, the urine leaves the suprapubic wound more directly and safely than it does by the long lacerated opening which forms the communication between the bladder and the perineal surface after the lateral operation for a large stone.

6. Because antiseptic dressing can be employed in the former operation and cannot be made available in the latter.

7. Because, in the suprapubic operation it is impossible to cut into the rectum, to inflict injury on the sexual organs, or to make an urethro-rectal or perineal fistula, any or all of which are liable to follow the lateral operation in a patient with a large stone.

The objections raised against the suprapubic method — the danger of opening the peritoneum and the risk of extravasation of urine around the base of the bladder—he does not regard as valid. The risk of extravasation, he says, is small, because it can only happen as the result of unnecessary and unwarrantable inteference with the tissues outside the bladder. As for the danger of lacerating the peritoneum, the experience of the modern operation demonstrates this danger as now virtually non-existent.

The writer then describes the technique of the operation at length, and adds tables of experience with the various methods. — *Weekly Medical Review.*

**Digestion of Infants.**—We hold that there is a presumption that an infant who passes regularly from four to six stools daily is passing a great deal of undigested food, and that so far the alimentation is imperfect. The wholesome fæces of a healthy breast-fed infant are of a bright gamboge-yellow, they are not unduly offensive, and they present very scanty traces of undigested casein. Now, in the majority of infants brought up on cow's milk, even diluted to the starvation point, the stools are distinctly offensive, and are largely made up of masses of white casein. When this occurs, to find fault with the liver and do nothing more than prescribe the inevitable grey powder appears to us the most absolute empiricism, and the antiseptic cleansing of feeding bottles and the banishment of tubes, excellent as these measures undoubtedly are, will not solve the difficulty in question.

How can the casein be dealt with ? There are several methods open to us. First, it may be predigested wholly or in part. The simplest and readiest way of accomplishing this has, in our experience, been brought about by the use of one of the preparations suggested by Sir William Roberts, which, made with fresh milk, is practically a partly digested milk gruel. But is doubtful whether this method, invaluable as we believe it to be in selected cases, ought to be tried as the first routine step in dealing with the difficulty in question.

Sir William Roberts, in his lectures at the College of Physicians, referred to an observation of his own made whilst feeding a healthy kitten exclusively on predigested food. Without obvious or marked departure from health, the kitten, nevertheless, in respect to body weight, fell behind another kitten fed on milk. It was suggested that some atrophy of unemployed glands might be responsible indirectly for this failure in nutrition. Now, in our problem, if we can get the infantile alimentary tract to digest for itself the casein masses, more will be gained than if the casein be predigested.

A simple, time-honored method, which we think ought always to be tried, is the dilution of the milk in varying proportions with barley-water, which, perhaps, in a mechanical way, facilitates the separation of the curd into more manageable masses. If this is found unsatisfactory, a very little malted food ought, we think, to be added to each bottle. That the whole character of the stools in respect to undigested casein may become altered after the adoption of this simple expedient we have satisfied ourselves again and again, as well as of the diminution in the actual number of the evacuations. But our pysiological friends protest that this is using farinaceous food, that the infant's pancreas and salivary glands are rudimentary, and that its economy is unequal to the conversion of starch To this objection there are several answers to be made. First, physiologically, it is by no means proved that the sole agents in the conversion of starch are the pancreas and the salivary glands, and experiments to prove the absolute incapacity of the infantile alimentary tract to convert a small amount of starch are not forthcoming. Again, in a good malted food a great part of the starch has been already converted and rendered soluble. But let it be granted that even after boiling there is a small amount of unaltered starch; if the net result is that the fæces of the child are more wholesome and less frequent, that it does not suffer from vomiting, acidity, and flatulence, and that it is gaining weight, what evidence is there that the small amount of starch has done harm?

The employment of the term "farinaceous" has, indeed, brought us more less under bondage in our directions as to infant feeding. To repeated meals of arrowroot, corn flour, baked flour, and the like, even when made with milk, and the term is applicable, and we are second to none in our condemnation of such methods of feeding infants; but a broad distinction ought to be drawn between them and the use of a very small quantity of malted soluble food added to milk. If even the malted foods be used in large relative proportion in early infancy, to the exclusion or great diminution of the quantity of fresh milk, we believe that serious risk is incurred in the direction of scurvy; and this is the more insidious because, with regard to the two tests which we have mentioned, the body weight may certainly increase, and the stools may be less offensive and less frequent than under a milk regimen. The proper use of the malted foods is that they should be employed in small quantity—not in any sense as a substitute for fresh milk, but as an aid to the digestion of the casein.

We have said nothing about the humanized milk and the cream-and-whey methods. Useful as these plans have unquestionably proved hitherto, they have been methods for the rich and not for the poor, and their discussion may be for the present deferred.—*London Lancet.*

**Quinine in Whooping Cough.**—Dr. Kohlonetz conceived, recently, the idea of injecting the remedy in solution with considerable force far back into the mouth against the walls of the pharynx. (*Deutsch. Med. Zeit.*) In this manner he believes that some of it gets into the larynx and that the large quantity of it comes into intimate contact with the epiglottis. a fact he considers of the utmost importance. He employs the following formula:

R. Quiniæ sulphatis........................grs. xxxij,

Acid. sulphur..............................*m.* xxxij,

Aq. destillat...........................ad. f. ℥ vj.

M.

During the first three days he injects a common glass syrngeful of this solution every two hours into the open mouth, the tongue being depressed by the mother with the aid of a spoon. The next four days these injections are made every three hours, and after that *ad libitum.* This dose is used in children from three to four years. In younger children the method and the execution are the same, the only difference being that the syringe is not filled quite so full.

The result has been remarkably good. As a rule, the whoop greatly diminished by the third day, and in almost all cases ceased totally by the end of the first week. Whenever the result was not so satisfactory, K. convinced himself that the treatment had not been carried out strictly according to directions, and he requests physicians to give the method a fair trial. As it can easily be executed, we advise our readers to do the same. Some children suffer so intensely from this disease that anything promising relief may well be worth a trial.—*Medical and Surgical Reporter.*

**Hemorrhoids.**—Mr. Banks, Liverpool: A patient of mine discovered for himself that the proper time to apply cold water to his hemorrhoids was when they were down, and not when he had drawn them up. So as soon as the bowel was evacuated he remained perfectly still, emptied the pan of the water-closet, and keeping the rush of water on with his right hand he threw it up with his left on to the everted mucous membrane. By this means he thoroughly cleansed his piles, and by the direct application of the cold water to them ensured contraction of their blood vessels and their complete retraction. Carefully drying the parts with a soft towel (and not using paper), he then washed his hands and was comfortable for the rest of the day.

This mystery I have communicated to a great many patients who have derived much comfort from it. Doubtless there is a certain want of dignity about the proceedings which renders its description a difficult matter to put in a text book, but that does not prevent its being a very valuable treatment, and one which makes all the difference between the useful and the useless employment of an excellent remedy.

If a man of middle age who is beginning to be bothered with hemorrhoids will be moderate in his stimulants, will use an enema of tepid water frequently, will apply cold water after his motion in the way I have described, and will then employ a little hamamelis ointment, he will almost infallibly save himself from getting so bad as to require operation.—*Epitome.*

**On Vaginal Extirpation of the Uterus.**—Dr. Brennecke, in the *Zeitschrift gur Geburtshulfe und Gynakologie*, states that he has removed the entire uterus through the vagina for cancer eighteen times, without a single death. His first principle is to make the parts as accessible to the surgeon's hand as possible throughout the operation. For this purpose, he has devised a special uterine clamp forceps, which takes up little room and grasps the tender and friable uterine tissues firmly yet safely. He takes special precaution against hemorrhage. He carefu ly cuts through the upper reflexion of the vagina, in front and behind, with a short bladed knife, Kuchenmeisters, and separates the cervix from the loose surrounding tissue. Then he isolates the denser tissue on the sides of the cervix, bearing the uterine artery and its branches. The operator then grasps the uterine appendages. Each appendage is ligatured by means of a strong S-shaped needle, resembling that devised by Olthauser, which introduces the thread with comparative facility. The uterus is then strongly retroflexed, and pressed closely against the posterior wall of the vagina; by this manipulation the vesico-uterine fold of peritoneum is most readily separated without injury to the bladder. An elastic ligature is then applied to the broad ligaments, and they are cut through. Dr. Brennecke neither drains Douglas's pouch, nor applies any sutures to its divided serous surfaces. An iodoform-glycerine plug sufficiently protects the escaping discharge from septic changes. After six or seven days the plug is removed, and the vagina is simply irrigated. Dr. Brennecke prefers total extirpation to amputation of the cervix, and even advocates vaginal extirpation for other incurable uterine diseases, in preference to abdominal section or oophorectomy. His monograph is of great importance, and his method of operating can only be thoroughly understood by a perusal of the same.

**New Method of Employing Electricity in Neuralgia.**—Dr. Adamkiewicz describes in the Polish journal *Prezlad lekarski* an exceedingly effective way of applying electricity for the relief of neuralgia of various kinds. The apparatus he uses is a constant current battery, the cathode being a concave metal plate lined with electrical carbon, which is saturated with chloroform. This is then applied over the painful spot, and a current, weak at first but gradually increasing in strength, passed. One or two applications of this kind were found, says the author, sufficient entirely to relieve the severest cases.—*Medical Reporter.*

**Burns.**—It is said the ideal dressing for a burn is the one that is thoroughly protective, hence comfortable, and one that can remain longest, viz., antiseptic. I think in the present state of our knowledge bismuth and absorbent cotton is the nearest approach to such a dressing.

*Mode of Application.*—The parts should be perfectly cleansed with carbolized water or listerine. I usually puncture any large vesications in second degree burns. Then if the burn be of small superficial extent powder it over with bismuth, over this a good thick layer of absorbent cotton, and over all a bandage. If the injury covers considerable extent, so as to render the too free use of bismuth dangerous, make a solution in water of the bismuth and paint it over the part. This last permits of uniform distribution of a minimum quantity. I have used this dressing in several cases of burn, and in one extensive scald of the leg, second and third degree, and so far have not witnessed any evidence of bismuth poisoning.

The results have been very satisfactory, in two or three cases scarcely any suppuration occuring. I have not used it in burns involving as much as one-fourth of the surface of the body, but think with care it may be used safely. A dressing of this kind promotes to the greatest degree healing by scabbing which is the method to be desired in burns. After removing the cotton, because of suppuration it may be, it is not necessary to remove the bismuth scab entirely, but to cleanse any point of suppuration and powder a little bismuth on, then re-apply fresh cotton. This method saves the surgeon much labor, the patient much pain, and does much to save life from septic absorption and suppurative exhaustion. Finally by promoting healing by scabbing instead of by granulation, it will do much to lessen subsequent contraction in burn cicatrices.—*Medical Progress.*

**Treatment of Bright's Disease.**—The author summarizes his treatment as follows:

An exclusive milk diet is absolutely essential. Milk acts as a complete aliment, and also as a diuretic. It is only occasionally that he allows a little meat or the yolk of eggs. Ordinary nitrogenous diet is proscribed.

Methodical and repeated dry frictions, massage, warm douches, and frequent hot-air baths, are indicated. Cold bathing and severe exercises are forbidden.

Residence in a dry and constant climate is best, while in winter or in a variable climate, the patient should go out little, and should exercise rather in an apartment with a constant temperature of from 64° to 68°. Iodide and the chloride of sodium should be administered in increasing doses up to the point of tolerance. When the albuminuria and anasarca have ceased, he gives the phosphate of soda and lime in doses of from gr. xv. to ʒ i. in twenty-four hours.

Methodical inhalations of oxygen should be employed.

Any kind of astringent is useless and harmful.—*L. Union Med.*—*Analeric.*

